# Excess Mortality by Individual and Spousal Education for Recent and Long-Term Widowed

**DOI:** 10.1093/geronb/gbab227

**Published:** 2021-12-08

**Authors:** Olof Östergren, Stefan Fors, Johan Rehnberg

**Affiliations:** 1 Aging Research Center, Karolinska Institutet and Stockholm University, Stockholm, Sweden; 2 Department of Public Health Sciences, Stockholm University, Stockholm, Sweden; 3 Center for Epidemiology and Community Medicine, Region Stockholm, Stockholm, Sweden

**Keywords:** Bereavement, Event history analysis, Register data, Social determinants of health

## Abstract

**Objectives:**

The loss of a spouse is followed by a dramatic but short-lived increase in the mortality risk of the survivor. Contrary to expectations, several studies have found this increase to be larger among those with high education. Having a spouse with high education is associated with lower mortality, which suggests that losing a spouse with high education means the loss of a stronger protective factor than losing a spouse with low education. This may disproportionately affect the high educated because of educational homogamy.

**Methods:**

We use Swedish total population registers to construct an open cohort of 1,842,487 married individuals aged 60–89 during 2007–2016, observing 239,276 transitions into widowhood and 277,946 deaths. We use Poisson regression to estimate relative and absolute mortality risks by own and spousal education among the married and recent and long-term widows.

**Results:**

We find an absolute increase in mortality risk, concentrated to the first 6 months of widowhood across all educational strata. The relative increase in mortality risk is larger in higher educational strata. Losing a spouse with high education is associated with higher excess mortality, which attenuates this difference.

**Discussion:**

When considering the timing and the absolute level of excess mortality, we find that the overall patterns of excess mortality are similar across educational strata. We argue that widowhood has a dramatic impact on health, regardless of education.

Widowhood is associated with an elevated risk of death that is dramatic and immediate but also short-lived (Moon et al., 2011; Shor et al., 2012). After the first year of widowhood, the death risk among the widowed is similar to that among the married (Moon et al., 2011). Losing a spouse entails an immediate and intense period of change and adaptation. The process of adapting may be especially important among older adults because they may rely on their partner not only for social and economic support, but also for performing household tasks and informal care work. Morbidity is more prevalent in old age, and older adults often rely on their partners for care needs and for help with adhering to treatment (Bertogg & Strauss, 2020). The risk of experiencing widowhood increases with age, especially among women, who live longer and tend to have older spouses. Among Swedish women aged 80, more than 40% are widows, making it the most common civil status in that age group (Statistics Sweden, 2016). Thus, widowhood has profound consequences for both living conditions and survival among older adults, the importance of which can be expected to increase with population aging.

There are good reasons to believe that education may be protective against excess mortality during bereavement. Education has in itself a strong association with health and mortality (Hayward et al., 2015; Lager & Torssander, 2012; Ross et al., 2012; Tillmann et al., 2017) and is associated with personal characteristics and cognitive skills (Batty et al., 2009; Mirowsky & Ross, 2003). Individuals with higher education tend to have access to more material and social resources (Huang et al., 2009) and are also more likely to use one type of resource to compensate for a deficit in another (Mirowsky & Ross, 2003; Östergren, 2018). Education may then help the individual to cope with the loss of a spouse and consequently reduce excess mortality among individuals with high education. Contrary to expectations, several studies that have evaluated whether education is a protective factor during bereavement have found that higher education is associated with higher excess mortality in widowhood relative to individuals with lower education (Boyle et al., 2011; Dabergott, 2021; Lusyne et al., 2001; Manor & Eisenbach, 2003). In this study, we shed light on this unexpected finding by considering the education of both the survivor and the deceased spouse while also considering the dramatic and short-lived nature of the excess mortality risk following the transition into widowhood using both relative and absolute measures of mortality risk.

## Spousal Education, Gender, and Excess Mortality in Widowhood

Marriage is in itself associated with lower mortality risk (Drefahl, 2012; Subramanian et al., 2008), but it also matters to whom the individual is married. Several studies have found that higher spousal education is associated with lower morbidity and mortality independent of own education, a finding that persists into old age (Brown et al., 2014; Jaffe et al., 2006; Kilpi et al., 2018; Skalická & Kunst, 2008; Spoerri et al., 2014; Torssander & Erikson, 2009). Furthermore, the educational achievement of spouses tends to be correlated, a phenomenon referred to as educational homogamy. There are several factors that may contribute to this pattern. Education regulates access to sites where spouses may meet, for example, universities (Blossfeld & Timm, 2003), and individuals in similar social positions are more likely to have shared interests and tastes (Bourdieu, 1984). The health advantage associated with high education might then partly be attributed to the fact that highly educated individuals are more likely to have a spouse who also has high education.

The difference in mortality risk by civil status can partly be due to positive selection into marriage. As Durkheim notes: “One has few chances of succeeding in founding a family if one does not possess certain qualities of health, fortune and morality” (Durkheim, 1897/2006, p. 190). However, spouses may also influence each other’s health by sharing material and nonmaterial resources. Married life entails some degree of mutual decision making, social and psychological support, and informal care that are likely to be beneficial for health (Egeland et al., 2002; Kiecolt-Glaser & Newton, 2001; Monden et al., 2003). As men and women typically take on different social roles in the relationship, it is possible that average contributions and rewards from such reciprocal exchanges differ between men and women. The loss of a partner may then have different consequences for men and women. Consequently, it is feasible that losing a partner may trigger gendered processes that ultimately affect the health of the surviving partner differently depending on gender. For example, men tend to have higher incomes while women tend to take on greater responsibility for the health of the family (Umberson, 1992; Umberson et al., 2010). Losing the bulk of the household income and losing concerted health management are both likely to affect the health of the surviving spouse, but in different ways. Umberson et al. (2010) have suggested that it is this loss of the wife’s active role in the health of her partner that underlies the greater increase in mortality associated with partner loss observed among men compared to among women (Moon et al., 2011; Umberson et al., 2010).

A spouse, especially a highly educated spouse, is then a protective factor for health. Losing a spouse with high education could imply the rescindment of a stronger protective factor, relative to the loss of a spouse with lower education. When considered together with educational homogamy, this could partly explain the finding that higher education is associated with a more pronounced death risk following the death of a spouse.

## The Scale and Timing of Excess Mortality in Widowhood

Studies assessing excess mortality in widowhood by education have typically compared mortality levels among the married and the widowed and estimated whether this difference is similar across educational strata (Boyle et al., 2011; Dabergott, 2021; Lusyne et al., 2001; Manor & Eisenbach, 2003; Martikainen & Valkonen, 1998; Sullivan & Fenelon, 2014). Although the mortality follow-up varies across studies, several of these studies compare all married to all widowed and do not differentiate between recent and long-term widowed (Boyle et al., 2011; Dabergott, 2021; Martikainen & Valkonen, 1998; Sullivan & Fenelon, 2014). An exception is the study by Manor and Eisenbach (2003), who isolated the educational differences in excess mortality after experiencing widowhood to the first 6 months of widowhood. Information on the timing of the interaction between education and widowhood may provide some clues for the plausibility of different explanations. If the interaction is limited to recent widows, this would corroborate acute processes, for example, acute emotional distress, support in performing daily tasks, or help with adhering to medical treatment. If, instead, the interaction can be detected among long-term widows, explanations that are focused on long-term processes may be more plausible, for example, decision making, health behaviors, and loss of resources.

Most previous studies have estimated excess mortality on a relative scale. An exception is the study by Martikainen and Valkonen (1998), who estimated excess mortality on both a relative and an absolute scale. They observed higher excess mortality among those with high education on the relative scale. However, due to the higher absolute level of mortality among those with lower education, they found more absolute excess mortality among the widowed in this group (Martikainen & Valkonen, 1998). Because higher education is associated with lower mortality, the baseline mortality before widowhood is lower in this group. A similar spike across educational strata in absolute excess mortality risk following widowhood then translates to smaller relative differences among persons with lower education and higher relative differences among persons with higher education. Considering both the relative and absolute measures of mortality may be informative for why the excess mortality in widowhood has been found to be more pronounced among the high educated.

## Aim

The aim of this study is to examine the association between both own and spousal education with mortality risks before and after the death of a spouse among men and women using Swedish population data. Specifically, we examine if educational differences in excess mortality during bereavement can be attributed to differences in the education of the deceased spouse. By considering the timing and scale of excess mortality, we aim to provide a better understanding of what types of processes are likely to contribute to educational differences in mortality risk during bereavement.

## Method

We combine data from several registers covering the Swedish population. These are collected for administrative purposes and contain information on demographic characteristics, socioeconomic position, and mortality. Each individual is issued a personal identification number at birth or immigration. This allows us to combine information from multiple registers but also to identify spouses.

We construct an open cohort encompassing all individuals aged 60–89 residing in Sweden at some point during 2007–2016. The age and period are chosen to observe the maximum number of events in ages where widowhood is common while maintaining consistency in data availability throughout the observation period. The registers are updated annually and only for individuals who are alive at the end of the year (except for the cause of death register). As a consequence, information on education was not available for the year that the person died. We set mortality follow-up to cover the years 2008–2017 with information on education being collected for the year prior. These are the most recent data available for analysis.

To capture the transition into widowhood, we only allow married individuals to enter the cohort. If the spouse died during the follow-up, they are classified as widowed and followed until they died themselves or aged out of the cohort. If both spouses died on the same day, they are both excluded because it is not possible to determine who was widowed. In the case of a divorce or emigration, the individual is right-censored and if the individual remarried after a period of widowhood, they are reclassified as married.

Studies on the risk of death following the death of a spouse typically exclude deaths among the widowed that happen in a short interval after the spouse or deaths in which the spouses died from common causes (Boyle et al., 2011; Lusyne et al., 2001). This is done to exclude deaths that are caused by the same event (e.g., the same accident or an infectious disease afflicting both spouses). However, spouses may share risk factors due to their common lifestyle and living conditions that may increase the risk of dying from the same cause, but not from the same event. To avoid misclassifying deaths, we link the data to the cause of death register and exclude deaths when both spouses died within 30 days from each other with at least one shared common cause of death, either as an underlying or contributing cause, that was either an infectious disease (ICD-10 Sections A–B) or an external cause of death (ICD-10 Sections S–Z). These conditions were met for eight out of 44,878 deaths. For a full report on the implications of using different exclusion criteria, see Supplementary Materials.

We collect information on education from the educational register for both spouses and construct three categories: compulsory (International Standard of Classification of Education [ISCED] 0–2), intermediate (ISCED 3–4), and tertiary (ISCED 5–6). Additionally, we collect information on age and sex from the total population register. Age and education are treated as time-varying covariates and are updated annually. However, the educational register is not updated after the age of 75 and we assume that the education remains stable at older ages.

We restrict the population to different-sex couples to facilitate interpretation and to avoid couples being included twice in the same model. We also exclude individuals for whom we are unable to achieve full linkage between the different registers and individuals with missing data on any covariate, either for the index person or the spouse, which led to the exclusion of 45,082 individuals (2.4%).

The final population comprises 1,842,487 individuals observed over 13,520,436 person-years, experiencing 239,276 transitions into widowhood and 277,946 deaths of which 44,878 were among the widowed. Because women tend to marry older men, there is a greater number of widowed women during the follow-up. The educational distribution is similar among men and women (see Table 1 for further sample characteristics).

**Table 1.** Summary Statistics of the Analytical Sample

		Men			Women		
Individuals (*n*)		970,213			872,274		
Widowed during follow-up (*n*)		74,578			164,698		
Person years (*n*)	Married	6,702,767			5,863,771		
	Widowed	271,055			682,843		
Deaths (*n*)	Married	156,584			76,484		
	Widowed	18,228			26,650		
Age (years)	Mean	71.0			70.6		
		Spousal education (%)					
		Com.	Int.	Ter.	Com.	Int.	Ter.
Education (%)	Compulsory	18	14	3	20	11	3
	Intermediate	10	20	9	14	19	8
	Tertiary	3	8	15	3	8	14

We first estimate the association between own and spousal education among married individuals and mortality. Second, we fit a series of regression models estimating mortality risks by own and spousal education and civil status (married/widowed). In the first model, we include civil status, education, and the interaction thereof. In the second model, we introduce spousal education and interaction terms between spousal education and civil status. Third, we divided the widowed into five groups based on the duration of widowhood (0–6, 7–12, 13–24, 25–36, and 37 or more months). We fit the same set of models using this more detailed timescale to differentiate between recent and long-term widows. Using the coefficients from these models, we estimate the absolute risk of mortality by widowhood status and duration of widowhood first by own education and then by spousal education.

The mortality risks are estimated using a series of Poisson regression models with number of deaths as the dependent variable and person-months at risk as the offset. To adjust for autocorrelation between repeated measurements of each individual, we use clustered standard errors defining each individual as a cluster. The Poisson model is a versatile option when the dependent variable of interest is an integer describing the number of events (Frome, 1983). We calculate both relative and absolute measures of mortality risk using the obtained coefficients: the incidence rate ratio, which describes the relative risk between exposure groups and estimated absolute death risks for different exposure groups, which are obtained in postestimation through the *lincom* command in Stata. All models are estimated for men and women separately and adjusted for age using both a linear and quadratic term and a linear term for year.

## Results

Table 2 presents associations between all-cause mortality, education, and spousal education for men and women separately expressed as incidence rate ratios with 95% confidence intervals (CIs). Having a spouse with higher education is associated with lower mortality risk, adjusting for own education and income. Differences in mortality risks by own education are more pronounced among women than among men, whereas the associations between spousal education and mortality risk were similar among men and women.

**Table 2.** Incidence Rate Ratios and 95% Confidence Intervals for All-Cause Mortality by the Education and Spousal Education Among Married Swedish Men and Women 2007–2016

		Education	Spousal education
Men	Compulsory	1.	1.
	Intermediate	0.91 (0.90–0.92)	0.95 (0.94–0.96)
	Tertiary	0.79 (0.78–0.80)	0.84 (0.83–0.85)
Women	Compulsory	1.	1.
	Intermediate	0.89 (0.88–0.91)	0.93 (0.92–0.95)
	Tertiary	0.72 (0.70–0.74)	0.81 (0.79–0.83)

*Note:* Adjusted for age, age squared, and year.

Next, we examine the relationships between own and spousal education and widowhood. Table 3 presents results from two models. Model 1 estimates the association between mortality and widowhood, own education, and the interaction between widowhood and education. Model 2 introduces spousal education and interaction terms between spousal education and widowhood. The first set of incidence rate ratios in Model 1 describes the mortality risk among the widowed relative to the mortality risk of the married. The ratios were 1.28 among men and 1.19 among women. The interaction terms between widowhood and education were above 1, indicating that the relative excess mortality following the death of a spouse was greater among those with tertiary education than among those with compulsory education. Among men, the ratio between the mortality risk of the widowed relative to the married was 1.28 among individuals with compulsory education and 1.34 (1.28 × 1.05 = 1.34) among individuals with tertiary education. The corresponding estimates among women were 1.19 among those with compulsory education and 1.38 (1.19 × 1.16 = 1.38) among those with tertiary education.

**Table 3.** Incidence Rate Ratios and 95% Confidence Intervals for All-Cause Mortality by Widowhood Status, Education, and Spousal Education Among Swedish Men and Women 2007–2016

		Model 1	Model 2
Men			
Civil status	Married	1.	1.
	Widowed	1.28 (1.25–1.30)	1.24 (1.21–1.27)
Education	Compulsory	1.	1.
	Intermediate	0.88 (0.87–0.89)	0.91 (0.90–0.92)
	Tertiary	0.73 (0.72–0.74)	0.79 (0.78–0.81)
	Widowed × Intermediate	1.03 (0.99–1.06)	1.00 (0.97–1.04)
	Widowed × Tertiary	1.05 (1.01–1.10)	1.00 (0.95–1.06)
Spousal education	Compulsory		1.
	Intermediate		0.95 (0.94–0-96)
	Tertiary		0.84 (0.83–0.85)
	Widowed × Intermediate		1.08 (1.05–1.12)
	Widowed × Tertiary		1.08 (1.02–1.14)
Women			
Civil status	Married	1.	1.
	Widowed	1.19 (1.17–1.21)	1.18 (1.15–1.20)
Education	Compulsory	1.	1.
	Intermediate	0.86 (0.85–0.87)	0.89 (0.87–0.90)
	Tertiary	0.65 (0.63–0.66)	0.71 (0.70–0.73)
	Widowed × Intermediate	1.04 (1.01–1.08)	1.04 (1.01–1.07)
	Widowed × Tertiary	1.17 (1.11–1.22)	1.13 (1.07–1.18)
Spousal education	Compulsory		1.
	Intermediate		0.93 (0.92–0.95)
	Tertiary		0.81 (0.79–0.83)
	Widowed × Intermediate		1.00 (0.96–1.03)
	Widowed × Tertiary		1.07 (1.02–1.12)

*Note:* Adjusted for age, age squared, and year.

Model 2 (Table 3) further shows the patterns of mortality risks among married and widowed by spousal education. The incidence rate ratios of the interaction terms between spousal education and widowhood were above 1 for both men and women, indicating that widowhood was associated with higher excess mortality when the deceased partner had higher education. When adjusting for spousal education, the finding that the excess mortality risk following the death of a spouse was larger among the high educated was attenuated among men. The interaction term in Model 1 was 1.05 (95% CI: 1.01–1.10), while the corresponding interaction term in Model 2 was 1.00 (95% CI: 0.95–1.06). Among women, both own and spousal education were associated with differences in mortality risks among the widowed, that is, the incidence rate ratios describing the interaction were similar across both models.

**Figure 1.**
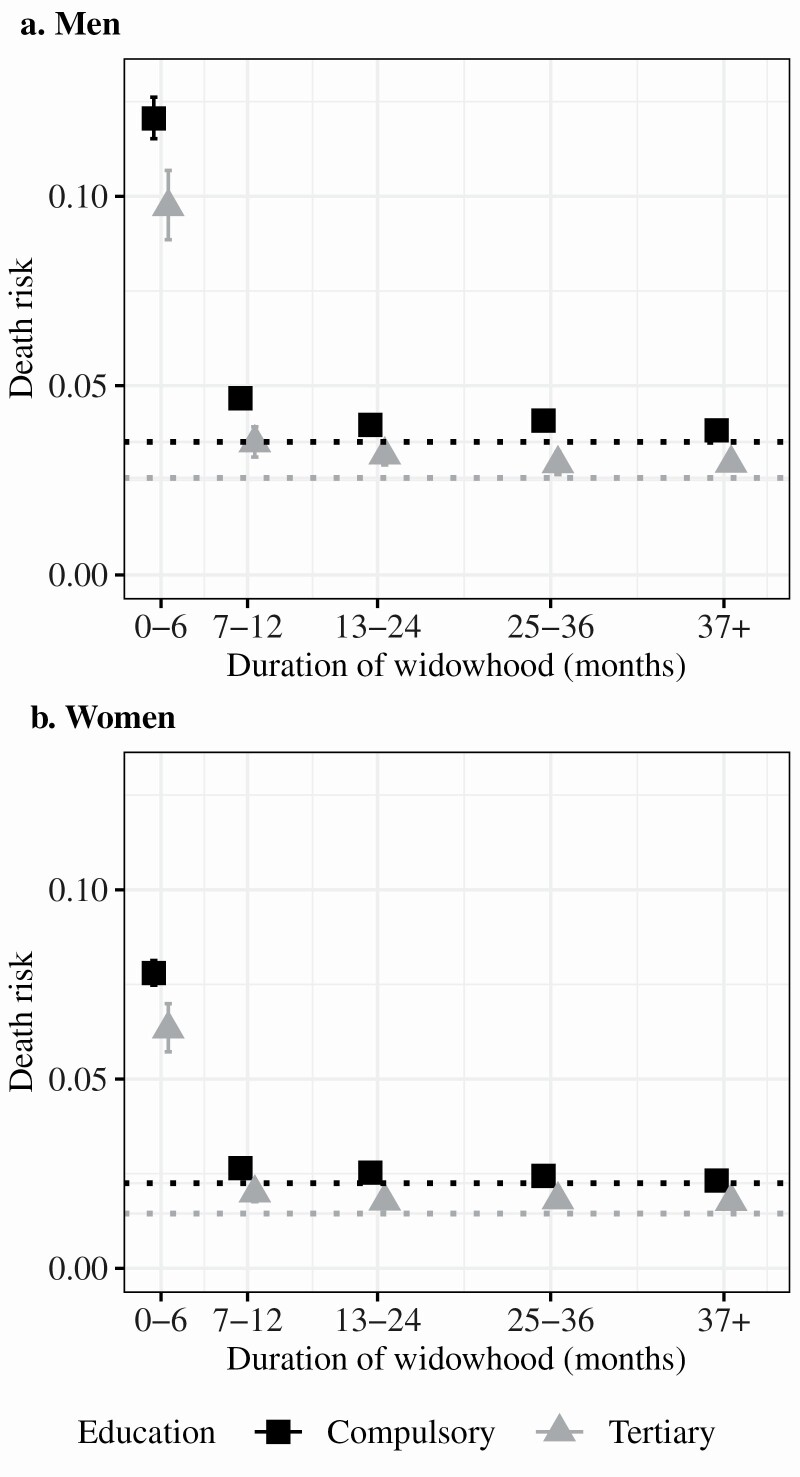
Estimated death risks and 95% confidence intervals for men (panel A) and women (panel B) by education and civil status were obtained from Poisson regression models adjusting for age, age squared, and year. The dotted lines indicate estimated mortality risks among the married with compulsory (black) or tertiary (gray) education, 2007–2016.

So far, we have not considered the short-lived and dramatic nature of the excess mortality among the widowed. Figure 1 depicts estimated 1-year death risks and 95% CIs by the duration of widowhood for individuals with tertiary education and individuals with compulsory education. The estimates are obtained using Poisson regression models where the association between widowhood and mortality and the interaction between education and widowhood are allowed to vary by duration of widowhood. The models are adjusted for age, age squared, and year. Individuals with intermediate education tend to have mortality levels in between those observed among the compulsory and tertiary educated, but are not represented in the graph. When estimating the death risks, we held age and year constant at their median values (77 and 2012, respectively), allowing only education and widowhood status to vary. The specific values at which age and year are held constant will influence the baseline death risk, and thereby the scale at which the risks are expressed, but not the pattern. The dotted lines indicate the death risks among the married with the corresponding education.

The excess mortality associated with widowhood is concentrated to the first 6 months and to a lesser extent the first year of widowhood whereas, after 13 months, the death risks are similar among the widowed and married. The dramatic increase in mortality risk was observed in both educational strata, and there was no tendency toward a larger increase among the high educated on the absolute scale (the estimated absolute mortality is presented in Supplementary Table 1). The mortality risk among long-term widowed men tended to remain somewhat higher than among the married in both educational groups. Among women, however, this pattern was only observed for women with tertiary education, whereas women with compulsory education who had been widowed for more than 2 years had a similar mortality risk as the married.

**Figure 2.**
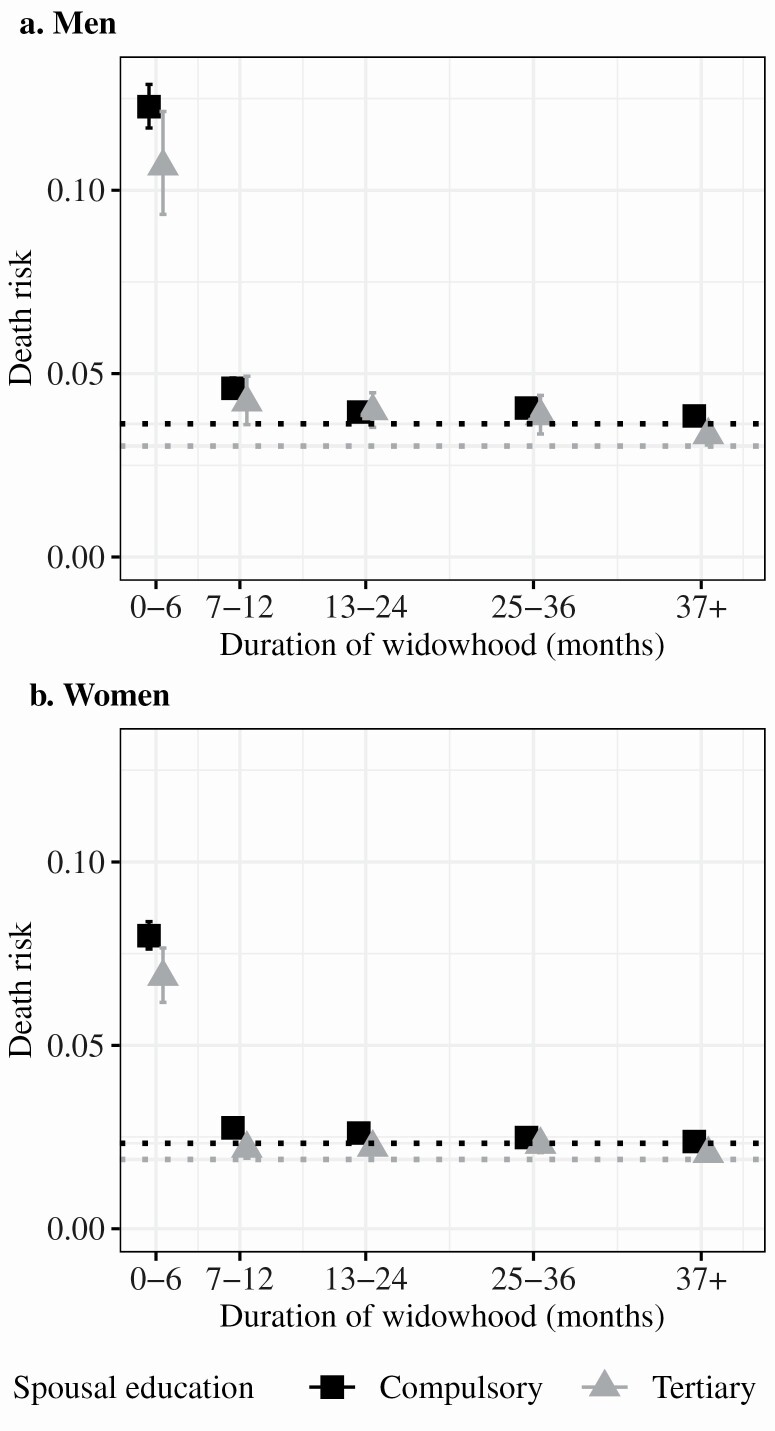
Estimated death risks and 95% confidence intervals for men (panel A) and women (panel B) by spousal education and civil status obtained from Poisson regression models adjusting for own education, age, age squared, and year. The dotted lines indicate estimated mortality risks among the married with compulsory (black) or tertiary (gray) spousal education, 2007–2016.

We then introduce spousal education into the models and estimate death risks by spousal education among the widowed, similar to those presented in Figure 1, only this time by spousal education instead of own education. The estimated death risks are presented in Figure 2 (see Supplementary Table 2 for the absolute death risks).

Among the married, the death risks for those with a spouse with tertiary education are lower compared to those with a spouse with compulsory education (see the dotted lines in Figure 2). While there was a tendency toward lower mortality among those who survived a spouse with higher education compared to those who survived a spouse with compulsory education in the first 6 months of widowhood, there were no clear systematic differences after the first year of widowhood.

### Sensitivity Analyses

We fitted several additional models that were adjusted for 5-year average disposable household income, the age of the spouse, hospitalizations in the years prior to widowhood, and children living at home. While several of these covariates were associated with mortality risk among the widowed, the observed patterns of excess mortality in widowhood by own and spousal education were robust across all specifications.

We tested for multicollinearity between own and spousal education by fitting a linear version of the Poisson model and estimating variance inflation factors (VIFs). The VIFs range from 1.39 to 1.83, indicating no issues with multicollinearity.

We tested for overdispersion by fitting negative binomial models. The alpha parameter was not significant, indicating that the data are not overdispersed. For a subset of the models, the negative binomial models did not converge. This was especially the case for models that do not consider the duration of widowhood. Because the negative binomial could not be used in all instances, we opted to consistently present results from Poisson models. We also estimated Cox-proportional hazards models, finding near-identical results.

## Discussion

### Summary of the Results

Widowhood was associated with an increase in mortality risk during the first year of widowhood and especially during the first 6 months. This association was observed for both women and men, but was stronger among men. After 1 year of widowhood, the mortality risks of the widowed and married were similar. The overall magnitude and timing of excess mortality were similar in all educational groups, although relative excess mortality was somewhat more pronounced among individuals with high education. Higher education was associated with lower mortality among the married and at all stages of widowhood while the association between higher spousal education and lower mortality risk was attenuated during widowhood.

### Spousal Education Is Associated With Mortality Among the Married—But Not the Widowed

In line with previous studies, we observe an association between higher spousal education and lower mortality risk, independent of own education, among the married. Both men and women experience a dramatic increase in excess mortality during the first 6 months of widowhood, but men experience larger excess mortality in widowhood compared to women, both on the absolute (Figures 1 and 2) and relative (Table 3) scales. The association between spousal education and mortality risk was attenuated 6 months after the transition into widowhood, which led to larger relative excess mortality at higher levels of spousal education among both men and women. Due to educational homogamy, this partly explains why the relative excess mortality associated with widowhood is larger among individuals with high education. We observe independent associations between own and spousal education and excess mortality in widowhood for women. Among men, adjusting for spousal education fully attenuated the association between excess mortality and own education, implying that spousal education was more important than own education. The observed gender difference in overall excess mortality and relative importance of spousal education implies that men rely more on their spouses than women do. This is in line with previous studies suggesting that women take greater responsibility for the health of the family (Umberson, 1992; Umberson et al., 2010). However, we also observed similarities among men and women in that both men and women experience a dramatic increase in excess mortality concentrated to the first 6 months of widowhood regardless of their own and spousal education.

There are several mechanisms through which spousal education may promote health. Spouses may pool their resources, make important decisions together, and develop habits that may have long-term implications for health. Spouses may also be a source of social and psychological well-being and help each other in managing health and daily activities, which may provide short-term benefits to health. Our findings point toward short-term processes and suggest that the health benefit of having a highly educated spouse is contingent on continuous contact with the spouse. We were not able to directly observe the underlying processes and it is possible that the processes generating the findings differ between men and women. It is also possible that the specific processes differ across stages of the life course. Long-term processes may be more important among younger couples while short-term processes are more important in older couples who rely on each other for direct support in managing health and performing daily activities.

### Similar Patterns of Timing and Scale of Excess Mortality in Widowhood Across Educational Strata

Our results indicate that among women with compulsory education, the widowed have a relative mortality risk of 1.19 to married women. This difference varies by duration of widowhood. The relative risk is 3.43 in the first 6 months and 1.09 after 3 or more years of widowhood. Similar patterns are observed across all educational strata and among men (these estimates were obtained from the coefficients in Figure 1, presented in Supplementary Table 3). While the relative differences are more pronounced among individuals with intermediate and tertiary education, the scale of the educational differences is substantially smaller compared to the dramatic spike and subsequent decline in excess mortality that follows the transition into widowhood in all educational strata. While it is not incorrect to compare the mortality of all widowed to the married, failing to take the dramatic differences by time into account may provide a misleading characterization of the event.

Previous studies on the association between education and the excess mortality associated with widowhood have focused on relative effect estimates (Boyle et al., 2011; Lusyne et al., 2001; Manor & Eisenbach, 2003). While we find that excess mortality was more pronounced among the highly educated on a relative scale, we find no clear systematic differences on an absolute scale. On the contrary, when considering the timing and magnitude of excess mortality, there are striking similarities in patterns of excess mortality in widowhood across educational strata. Regardless of educational attainment, the loss of a spouse is followed by a dramatic increase in mortality risk. Relative differences tend to be smaller when the baseline risk is higher (Eikemo et al., 2009) and because of the dramatic shift in absolute mortality risks immediately following the death of a spouse, relative differences between educational groups narrow. This suggests that educational differences in excess mortality associated with widowhood on a relative scale in part result from a combination of a lower mortality risk before widowhood and a similar increase in mortality across all educational strata that shift the scale on which the relative differences are expressed. Several of the explanations that have been discussed as contributing to the relative educational difference in excess mortality following widowhood have focused on the ways in which the association between widowhood and mortality is *different* across educational strata. The results from this study instead suggest that scholars should focus on why excess mortality in widowhood is *similar* across educational strata.

### Limitations

We observed the Swedish population over a 10-year period when 239,276 new widowhoods and 277,946 deaths occurred. We divided the population by both own and spousal education and by duration of widowhood and fitted models that included several sets of interaction terms. To avoid cells with a small number of individuals and few events, we categorized both duration of widowhood and education into broad categories. These categorizations were made based on both substantive and technical considerations.

We divided duration of widowhood into five categories (0–6, 7–12, 13–24, 25–36, and 37 or more months). While these capture periods of widowhood when the mortality risk is different, the mortality risk varies within each period, especially during the first 6 months of widowhood (Supplementary Figure 1). The results presented in Figures 1 and 2 likely represent an underestimation of the increase in mortality risk immediately following widowhood.

Education was modeled using three categories: compulsory, intermediate, and tertiary. The data comprised birth cohorts born up to 40 years apart during a period when average educational achievement increased steadily, especially among women. While this is a comparatively crude measure of education, it allows us to meaningfully analyze a wider range of birth cohorts with similar measures of education for both spouses. Because we are modeling both own and spousal education, we considered the distribution of the different combinations of educational achievement within couples. Couples with large educational disparities are uncommon (Table 1). Using few categories results in fewer cells with small cell counts and more stable estimates. Furthermore, this scale has been used in prior studies and using this categorization allowed us to relate the findings to the wider literature and discuss how different modeling strategies can influence the interpretation of results.

There is variation within the educational categories in terms of the number of years spent in education and the specific skills that are acquired. However, the categorization used distinguishes between three distinct forms of educational institutions in Sweden (see Halldén (2008) for a detailed description of how the ISCED scale is related to the Swedish educational system). Each step in the used scale requires the individual to actively transition into a separate educational institution and pursue further education for several years. Each additional step also expands the options in the labor market. Still, it is possible that the results would differ using a different classification scheme. For an expanded discussion on the coding of education, see Supplementary Materials.

Previous studies on educational differences in excess mortality during bereavement have been conducted using data from several comparatively egalitarian high-income welfare states including Sweden (Dabergott, 2021), Scotland (Boyle et al., 2011), Belgium (Lusyne et al., 2001), Israel (Manor & Eisenbach, 2003), and Finland (Martikainen & Valkonen, 1998). While the overall empirical findings seem to be robust across these social contexts, it is possible that the national context—for example, in terms of social inequalities, welfare arrangements, and gender ideology—modifies the processes that shape excess mortality in widowhood. Research in a wider range of contexts is needed in order to disentangle the importance of specific individual and contextual factors.

## Concluding Remarks

We set out to examine whether spousal education could explain educational differences in excess mortality following the death of a spouse. We found that spousal education in part accounted for why relative excess mortality in widowhood tends to be more pronounced among the high educated. However, the more striking finding was not that the excess mortality following the death of a spouse is different across educational strata, but that it is similar. Regardless of education, losing a spouse is followed by a dramatic short-term increase in mortality that results in a temporary reduction in relative, but not absolute, educational differences in mortality. Our results are consistent with the understanding that widowhood is a traumatic event that has a ubiquitous presence in the life of the widowed regardless of the educational qualifications of the individual or of the spouse.

## Supplementary Material

gbab227_suppl_Supplementary_Materials
