# Supplementary material for: Excess Mortality by Individual and Spousal Education for Recent and Long-Term Widowed
Source: J Gerontol B Psychol Sci Soc Sci. 2021 Dec 8;77(5):946–55. doi: 10.1093/geronb/gbab227 (PMC9071383; doi:10.1093/geronb/gbab227)
Supplement: gbab227_suppl_Supplementary_Materials [file gbab227_suppl_supplementary_materials.pdf]

# Excess mortality by individual and spousal education for recent and long-term widowed

*Supplementary materials*

Table S1. Estimated one-year death risks by education and widowhood obtained from Poisson regression models adjusting for age, age squared and year. Swedish men and women 2007—2016. Presented in Figure 1.

| | | | Duration of widowhood (months) | | | | |
|---|---|---|---|---|---|---|---|
| | Education | Married | 0—6 | 7—12 | 13—24 | 25—36 | 37+ |
| Men | Compulsory | 0.035 | 0.121 | 0.047 | 0.040 | 0.041 | 0.038 |
| | Intermediate | 0.031 | 0.107 | 0.042 | 0.037 | 0.036 | 0.036 |
| | Tertiary | 0.026 | 0.097 | 0.035 | 0.032 | 0.029 | 0.030 |
| Women | Compulsory | 0.022 | 0.078 | 0.026 | 0.025 | 0.024 | 0.023 |
| | Intermediate | 0.019 | 0.068 | 0.026 | 0.023 | 0.021 | 0.021 |
| | Tertiary | 0.014 | 0.063 | 0.020 | 0.018 | 0.018 | 0.018 |

Note: The coefficients and standard errors used to estimate the death risks are presented in Table S3.

Table S2. Estimated one-year death risks by spousal education and widowhood obtained from Poisson regression models adjusting for education, age, age squared and year. Swedish men and women 2007—2016. Presented in Figure 2.

| | | | Duration of widowhood (months) | | | | |
|---|---|---|---|---|---|---|---|
| | Spousal education | Married | 0—6 | 7—12 | 13—24 | 25—36 | 37+ |
| Men | Compulsory | 0.036 | 0.123 | 0.046 | 0.040 | 0.041 | 0.038 |
| | Intermediate | 0.034 | 0.120 | 0.052 | 0.041 | 0.043 | 0.039 |
| | Tertiary | 0.030 | 0.107 | 0.042 | 0.040 | 0.038 | 0.033 |
| Women | Compulsory | 0.023 | 0.080 | 0.027 | 0.026 | 0.025 | 0.024 |
| | Intermediate | 0.022 | 0.075 | 0.025 | 0.024 | 0.024 | 0.022 |
| | Tertiary | 0.019 | 0.069 | 0.022 | 0.022 | 0.023 | 0.020 |

Note: The coefficients and standard errors used to estimate the death risks are presented in Table S4.

Table S3. Coefficients and standard errors used to estimate absolute death risks and confidence intervals presented in Figure 1a-b and Table S1.

| | | Men | | Women | |
|---|---|---|---|---|---|
| | | Coef. | SE | Coef | SE |
| Civil status | Married | 0 | Ref. | 0 | Ref. |
| Widowed | 0—6 months | 1.233 | 0.023 | 1.244 | 0.021 |
| | 7—12 months | 0.284 | 0.028 | 0.163 | 0.025 |
| | 13—24 months | 0.121 | 0.023 | 0.117 | 0.019 |
| | 25—36 months | 0.149 | 0.025 | 0.083 | 0.021 |
| | 37+ months | 0.084 | 0.017 | 0.031 | 0.013 |
| Education | Compulsory | 0 | Ref. | 0 | Ref. |
| | Intermediate | -0.124 | 0.006 | -0.154 | 0.008 |
| | Tertiary | -0.315 | 0.007 | -0.440 | 0.011 |
| Education × Civil status | | | | | |
| Int. × Widowed | 0—6 months | 0.007 | 0.038 | 0.023 | 0.037 |
| | 7—12 months | 0.017 | 0.046 | 0.120 | 0.043 |
| | 13—24 months | 0.043 | 0.036 | 0.041 | 0.032 |
| | 25—36 months | 0.013 | 0.040 | 0.001 | 0.036 |
| | 37+ months | 0.056 | 0.027 | 0.057 | 0.022 |
| Ter. × Widowed | 0—6 months | 0.100 | 0.053 | 0.230 | 0.056 |
| | 7—12 months | 0.024 | 0.065 | 0.155 | 0.068 |
| | 13—24 months | 0.092 | 0.050 | 0.088 | 0.051 |
| | 25—36 months | -0.013 | 0.058 | 0.139 | 0.054 |
| | 37+ months | 0.057 | 0.037 | 0.168 | 0.032 |
| Controls | Age | 0.067 | 0.006 | -0.070 | 0.008 |
| | Age sq | 0.000 | 0.000 | 0.001 | 0.000 |
| | Year | -0.019 | 0.001 | -0.007 | 0.001 |
| | Intercept | 27.030 | 1.710 | 8.015 | 2.310 |

Table S4. Coefficients and standard errors used to estimate absolute death risks and confidence intervals presented in Figure 2a-b and Table S1.

| | | Men | | Women | |
|---|---|---|---|---|---|
| | | Coef. | SE | Coef | SE |
| Civil status | Married | 0 | Ref. | 0 | Ref. |
| Widowed | 0—6 months | 1.219 | 0.025 | 1.233 | 0.024 |
| | 7—12 months | 0.237 | 0.031 | 0.166 | 0.028 |
| | 13—24 months | 0.085 | 0.024 | 0.114 | 0.021 |
| | 25—36 months | 0.112 | 0.027 | 0.065 | 0.023 |
| | 37+ months | 0.058 | 0.018 | 0.020 | 0.015 |
| Education | Compulsory | 0 | Ref. | 0 | Ref. |
| | Intermediate | -0.055 | 0.006 | -0.069 | 0.008 |
| | Tertiary | -0.182 | 0.008 | -0.209 | 0.011 |
| Education × Civil status | | | | | |
| Int. × Widowed | 0—6 months | 0.036 | 0.041 | 0.005 | 0.038 |
| | 7—12 months | 0.169 | 0.047 | -0.036 | 0.045 |
| | 13—24 months | 0.103 | 0.038 | -0.023 | 0.034 |
| | 25—36 months | 0.112 | 0.042 | 0.014 | 0.037 |
| | 37+ months | 0.070 | 0.028 | -0.003 | 0.022 |
| Ter. × Widowed | 0—6 months | 0.040 | 0.067 | 0.058 | 0.057 |
| | 7—12 months | 0.095 | 0.080 | -0.021 | 0.067 |
| | 13—24 months | 0.189 | 0.061 | 0.046 | 0.050 |
| | 25—36 months | 0.127 | 0.070 | 0.129 | 0.054 |
| | 37+ months | 0.037 | 0.044 | 0.058 | 0.033 |
| Spousal education | Compulsory | 0 | Ref. | 0 | Ref. |
| | Intermediate | -0.098 | 0.006 | -0.121 | 0.008 |
| | Tertiary | -0.237 | 0.008 | -0.342 | 0.012 |
| Spousal education × Civil status | | | | | |
| Int. × Widowed | 0—6 months | -0.006 | 0.040 | 0.018 | 0.039 |
| | 7—12 months | -0.031 | 0.047 | 0.132 | 0.045 |
| | 13—24 months | 0.005 | 0.038 | 0.043 | 0.034 |
| | 25—36 months | -0.022 | 0.042 | -0.013 | 0.037 |
| | 37+ months | 0.037 | 0.028 | 0.052 | 0.022 |
| Ter. × Widowed | 0—6 months | 0.072 | 0.059 | 0.203 | 0.061 |
| | 7—12 months | -0.039 | 0.072 | 0.167 | 0.074 |
| | 13—24 months | 0.003 | 0.057 | 0.069 | 0.055 |
| | 25—36 months | -0.080 | 0.065 | 0.080 | 0.059 |
| | 37+ months | 0.031 | 0.041 | 0.140 | 0.035 |
| Controls | Age | 0.064 | 0.006 | -0.075 | 0.008 |
| | Age sq | 0.000 | 0.000 | 0.001 | 0.000 |
| | Year | -0.017 | 0.001 | -0.006 | 0.001 |
| | Intercept | 24.187 | 1.716 | 7.303 | 2.310 |

Figure S1. Age standardized mortality rates and 95% confidence intervals among the married and by month of widowhood.

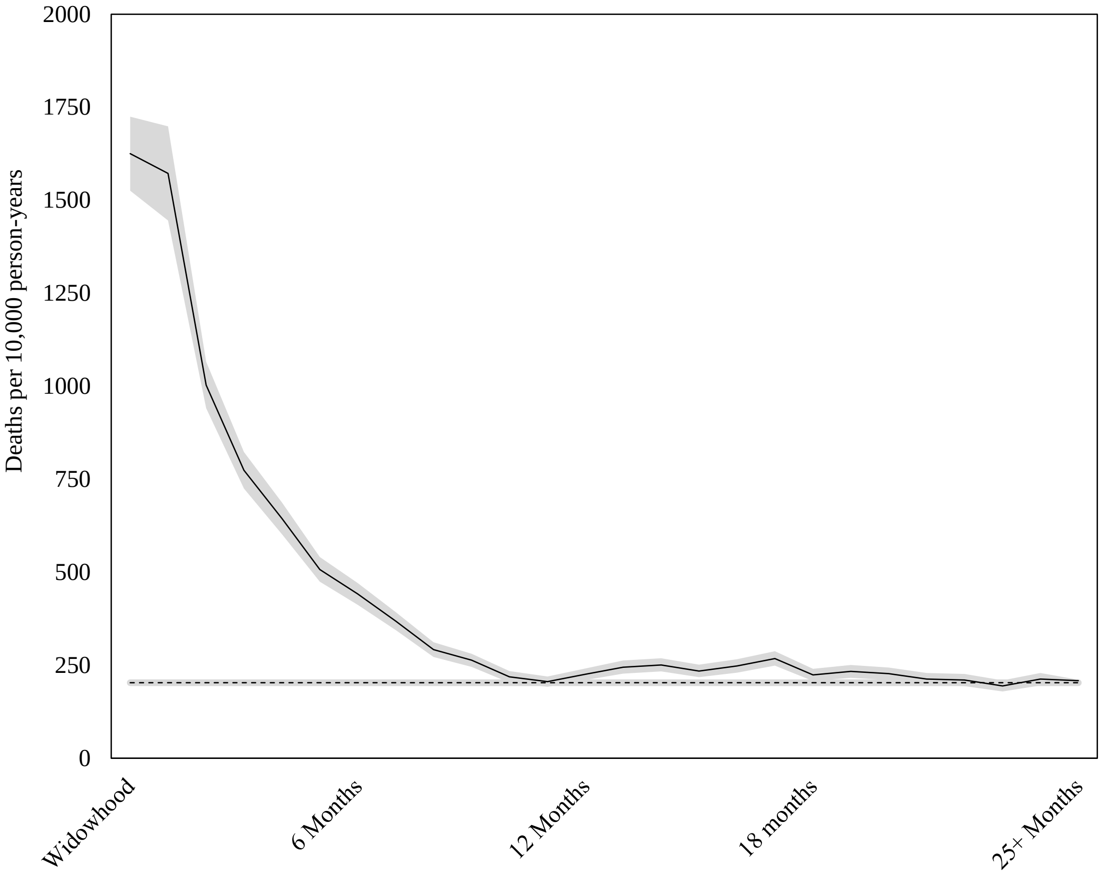


Notes: The dashed line denotes the mortality rate among the married. The age distribution of the complete sample was used as the standard population. Confidence intervals were calculated using the Keyfitz (1966)-method.

References

Keyfitz, N. (1966). Sampling variance of standardized mortality rates. *Human Biology*, 38(3), 309-317.

*Supplementary report on the importance of different exclusion criteria for deaths among the widowed*

Studies on mortality risks following widowhood typically attempts to exclude deaths among survivors that were caused by the same event that led to the death of the spouse. For example, if a married couple are in the same car accident, one may die before the other. In that case, the death should not be classified as a death in widowhood. In order to avoid misclassifying deaths, studies have adopted several strategies. These involve excluding deaths in close temporal proximity (Lusyne *et al.* 2001), deaths from common causes (Martikainen and Valkonen 1996) or a combination of both (Boyle *et al.* 2011). However, even if the spouses die from the same causes, it is not certain that these are the result of the same event. While the same accident and infectious disease can afflict both spouses, this is likely not the case for non-communicable diseases. Since these comprise the majority of deaths and the mortality risk following widowhood is concentrated to the period immediately following the widowhood event, excluding deaths without considering the specific causes may result in underestimating the association between widowhood and mortality risk.

We performed a series of analysis using different criteria for excluding deaths. First, we describe how many deaths are excluded by the different criteria and run a series of regression models, examining the short-term, long-term and overall associations between widowhood and mortality using the different criteria. The criteria used were no exclusions, deaths within 7 days, deaths within 30 days, all deaths from common causes, deaths from common causes within 7 days, deaths from common causes within 30 days and finally, deaths from common external causes or infectious diseases within 30 days.

Second, we present the distribution of common causes of death across the different sections of ICD-10. In order to assess common causes, we scanned the death certificates of both spouses for any matching causes in either the underlying or any of the 25 contributing causes in the Swedish cause of death register.

Table S4. Number of excluded deaths and overall, short- and long-term associations between widowhood and mortality using different exclusion criteria. Coefficients and standard errors obtained from Poisson regression adjusting for age, age squared, year and sex among Swedish men and women aged 60—90, 2007—2016.

| | | | | Duration of widowhood (months) | | | |
|---|---|---|---|---|---|---|---|
| | Excluded | Widowed | | 0—6 | | 7+ | |
| Criteria | deaths (n) | Coef. | SE | Coef. | SE | Coef. | SE |
| No exclusions | 0 | 0.237 | 0.006 | 1.270 | 0.012 | 0.134 | 0.006 |
| All deaths within 7 days | 284 | 0.230 | 0.006 | 1.228 | 0.012 | 0.134 | 0.006 |
| All deaths within 30 days | 1087 | 0.211 | 0.006 | 1.094 | 0.013 | 0.134 | 0.006 |
| All common causes | 6863 | 0.078 | 0.006 | 1.075 | 0.014 | -0.016 | 0.006 |
| Any common cause within 7 days | 67 | 0.235 | 0.006 | 1.260 | 0.012 | 0.134 | 0.006 |
| Any common cause within 30 days | 224 | 0.232 | 0.006 | 1.237 | 0.013 | 0.134 | 0.006 |
| Common external causes or infectious diseases within 30 days | 8 | 0.236 | 0.006 | 1.269 | 0.012 | 0.134 | 0.006 |

Note: The married were the reference category.

The number of deaths excluded vary by the criteria. Using a single criterion, i.e. only considering the timing of the death or common causes, excluded more deaths compared to using a combination. About 75% of the deaths occurring within 7 or 30 days of widowhood were not due to common causes. This indicates that deaths of spouses occurring in close proximity are predominately due to different causes.

The overall difference in mortality between the widowed and married were similar across most exclusion criteria, except for when excluding all common causes, attenuating the excess risk

among the widowed from 27% ($e^{0.237}$=1.267) to 8% ($e^{0.078}$=1.081), likely due to the large number of deaths excluded among the widowed, 6,863 out of 44,874 (15.3%), while no deaths are being excluded among the married. Excluding all deaths from common causes also attenuated the excess mortality risk among both recent and long-term widowed. The other exclusion criteria only exclude deaths among short term-widowed and predictably did not influence the excess risk estimated among long-term widowed. The magnitude of the estimated mortality following the death of a spouse depends on how many deaths among short-term widows are excluded. Since around 75% of deaths within the first months of widowhood are due to causes that did not play a part in the death of the spouse, excluding all deaths during this period is likely an underestimation of the spike in mortality. Since around 25% of the deaths during this period *are* caused by common causes, not excluding any deaths may overestimate the spike since both spouses may have died as the result of the same event. Whether or not this is plausible depends on the specific cause of death that the spouses have in common.

Table S5. Causes of death reported on both spouses death certificates by ICD section and duration of widowhood.

| Section | | All | 30 Days | 7 Days |
|---|---|---|---|---|
| A—B | Certain infectious and parasitic diseases | 96 | 2 | |
| C | Neoplasms | 766 | 24 | 4 |
| D | Diseases of the blood and blood-forming organs and certain disorders involving the immune mechanism | 19 | 1 | |
| E | Endocrine, nutritional and metabolic diseases | 366 | 6 | 1 |
| F | Mental, Behavioral and Neurodevelopmental disorders | 528 | 26 | 13 |
| G | Diseases of the nervous system | 120 | 8 | 1 |
| H | Diseases of the eye, adnexa the ear and mastid processes | | | |
| I | Diseases of the circulatory system | 4972 | 172 | 52 |
| J | Diseases of the respiratory system | 1117 | 31 | 13 |
| K | Diseases of the digestive system | 36 | 2 | |
| L | Diseases of the skin and subcutaneous tissue | 8 | | |
| M | Diseases of the musculoskeletal system and connective tissue | 2 | | |
| N | Diseases of the genitourinary system | 173 | 4 | |
| O | Pregnancy, childbirth and the puerperium | | | |
| P | Certain conditions originating in the perinatal period | | | |
| Q | Congenital malformations, deformations and chromosomal abnormalities | | | |
| R | Symptoms, signs and abnormal clinical and laboratory findings, not elsewhere classified | 987 | 35 | 19 |
| S—U | Injury, poisoning and certain other consequences of external causes | 25 | 5 | 2 |
| V—Y | External causes of mortality | 67 | 7 | 4 |
| Z | Factors influencing health status and contact with health services | 21 | | |

Table S4 displays the number of times specific causes of deaths were reported on both spouses' death certificates, either as an underlying or contributing cause of death, by each section of the 10th revision of the International Classification of Diseases (ICD-10). In most cases, spouses had one cause in common, but in 1,843 cases, the spouses had two or more causes in common. Therefore, number of times we identified the same cause of death on both death certificates is larger than the total number of deaths excluded. Most of the matches (88%) were identified in the contributing causes. Most of the common causes were non-communicable diseases. Together, neoplasms (C), diseases of the circulatory system (I) and diseases of the respiratory system (J) accounted for 70% of the instances where the same cause of death was reported for both spouses. About 11% of common causes were found in section R (symptoms, signs and abnormal clinical and laboratory findings, not elsewhere classified). This category is common in cases of multimorbidity when the exact causes and progression of the death is difficult to ascertain. This is common in older persons such as the population under consideration here (aged 60—90). In these instances, it is uncertain whether the actual cause of death is the same.

Excluding deaths using a single criteria, either the duration since the death of a spouse or whether the spouses had common causes of death, may lead to underestimating the association

between widowhood and mortality risk, especially among short-term widows. 75% of deaths happening shortly after the death of a spouse are due to different causes and the 70% of the common causes are due to cancer, circulatory disease or respiratory disease. Researchers that estimate the death risk following the death of a spouse should consider both the timing and the cause of death, both underlying and contributing causes. The exact criteria of exclusion may depend on the specific research question.

References

Boyle, P.J., Feng, Z. and Raab, G.M. (2011) Does widowhood increase mortality risk? Testing for selection effects by comparing causes of spousal death, *Epidemiology*. 1–5.

Lusyne, P., Page, H. and Lievens, J. (2001) Mortality following conjugal bereavement, Belgium 1991-96: The unexpected effect of education, *Population Studies*. 55, 3, 281–9.

Martikainen, P. and Valkonen, T. (1996) Mortality after death of spouse in relation to duration of bereavement in Finland., *Journal of Epidemiology & Community Health*. 50, 3, 264–8.

*Supplementary report on the coding of education*

In this study, we classify the highest achieved education by using three categories based on the ISCED-97 scale: compulsory education (ISCED 0—2), intermediate education (ISCED 3—4) and tertiary education (ISCED 5—6). In general, when fitting regression models, fewer categories give more robust estimates compared to using more categories, since fewer categories will contain more observations. Further, by using fewer educational categories, we allow for more complexity in other parts of the model, for example in differences in mortality depending on the duration of widowhood. On the other hand, when using fewer categories we risk obscuring important within-group variation. It is therefore important to account for important methodological and substantive aspects, as well as the specific national and historical context. Below, we provide an expanded discussion on how we considered these aspects in the relation to this study.

We model both own and spousal education, effectively estimating the association between the education of a couple and mortality in nine different groups (i.e., all possible combinations of own and spousal education). Using, for example, four educational categories instead of three, would result in 16 groups, and if we used the full ISCED, we would need to estimate mortality using 49 groups. Moreover, the issue of cells with small numbers become more pronounced because of educational homogamy, i.e., large educational differentials within couples are uncommon.

We also consider the coding schemes used by previous studies that estimate the relationship between widowhood and mortality by education. In this study, we find similar results to other studies when modeling the data in a similar way, without accounting for absolute levels and the timing of mortality during widowhood. This point is important since it shows that our data displays similar patterns to other data when modeled similarly, but that the interpretation of the results shifts when the timing is modeled and the absolute scale is considered. In turn, this means that the modeling strategy matters for the interpretation of the importance of education in relation to mortality in widowhood. This point is easier to make when the studies are comparable in terms of the educational classification used.

The three-level ISCED scheme used matches fairly well with the structure of the Swedish educational system. The compulsory group consists of all individuals that have lower secondary schooling or lower as their highest education (ISCED 0—2). During compulsory school, there were no tracking in the Swedish system for the cohorts under consideration. In more recent years, there has been a development towards marketization which has led to more diversity, both in terms of quality and content in Swedish schools (Wennström, 2020). The intermediate group consists of individuals with upper secondary schooling (ISCED 3—4). These are equivalent to codes starting with 3* (vocational) and 4* (academic) upper secondary schooling using the national Swedish classification, SUN (Svensk utbildningsnomenklatur). However, graduates of both the 3* and 4*-programs are eligible to advance to tertiary education and we therefore consider this group as having completed upper secondary schooling (though individuals with vocational degrees are less likely to advance to tertiary education). The SUN classification differentiates between licentiate and doctorate degrees which the ISCED does not. Apart from that, there are only small differences between the SUN and ISCED in tertiary education (ISCED 5—6). The relationship between the Swedish educational system and the ISCED scheme has been described in detail by Halldén (2008).

We argue that the three levels of education used in this study represent distinctive categories of education, where the differences between categories are more meaningful than the differences within categories. Each additional step in the scale we use require the individual to transition into a separate educational institution and pursue further education for several years. Each additional step also expands the options on the labor market. However, there is likely remaining variation in mortality within each educational category, both by levels within the categories, in particular in the group with tertiary education, and potentially by field of study, which may shape both labor market chances and specific competences that may be helpful in managing health.

References


Halldén, K. (2008). The Swedish educational system and classifying education using the ISCED-97. In Schneider, S. L. (Ed.) *The International Standard of Classification of Education (ISCED-97). An Evaluation of Content and Criterion Validity in 15 European Countries* (253-267). MZES.

Wennström, J. (2020). Marketized education: How regulatory failure undermined the Swedish school system. *Journal of Education Policy*, 35(5), 665-691.
